# Bidirectional Crosstalk Between Vulnerable Coronary Plaque and Coronary Microvascular Dysfunction: A Narrative Review

**DOI:** 10.3390/biomedicines14102179

**Published:** 2026-09-25

**Authors:** Majid Anwar, Anthony P. Davenport, Stephen P. Hoole

**Affiliations:** 1 Department of Cardiology, Royal Papworth Hospital, Cambridge CB2 0AY, UK; s.hoole@nhs.net; 2 Experimental Medicine and Immunotherapeutics, Department of Medicine, University of Cambridge, Addenbrooke’s Hospital, Cambridge CB2 0QQ, UK; apd10@medschl.cam.ac.uk

**Keywords:** ischaemia, vulnerable plaque, coronary microvascular dysfunction, heart failure with preserved ejection fraction, coronary to microcirculatory biological crosstalk

## Abstract

Coronary microvascular dysfunction (CMD) is a major contributor to myocardial ischaemia and angina, both in the presence and absence of obstructive coronary artery disease, yet it remains underdiagnosed and therapeutically challenging. The mechanistic links between CMD, myocardial ischaemia, and major adverse cardiovascular events (MACE) are incompletely understood. Emerging mechanistic, imaging, and clinical evidence suggests that CMD and vulnerable plaque (VP) could be connected through shared inflammatory, metabolic, and haemodynamic pathways. This review synthesises data across these domains to propose a bidirectional plaque–microcirculatory crosstalk framework, whereby each process may amplify the other. Evidence indicates that plaque-derived inflammatory mediators and haemodynamic disturbances can impair microvascular function, while CMD-related flow abnormalities may, in turn, accelerate plaque inflammation, progression, and destabilisation. Furthermore, distinct CMD endotypes (e.g., structural or functional) have different underlying pathophysiology and present with different phenotypes. Identifying mediators of coronary–microvascular crosstalk presents an opportunity to refine cardiovascular risk stratification and uncover novel therapeutic targets. Current anti-inflammatory therapies have shown variable effects, and CMD-directed vasomodulatory interventions have produced mixed results, partly due to limited endotype stratification. Emerging approaches, including coronary sinus reducer therapy and precision anti-inflammatory strategies, may offer dual benefits by improving CMD and stabilising vulnerable plaques. This review examines the bidirectional crosstalk hypothesis, aiming to provide an integrative framework linking epicardial atherosclerosis and microvascular pathology and may guide future phenotype-specific mechanistic and interventional studies aimed at reducing residual cardiovascular risk.

## 1. Introduction

The clinical importance of coronary microvascular dysfunction (CMD) in patients with stable angina is now well recognised; over 30% of patients with anginal symptoms have no significant epicardial stenoses, rising to 50% in females [1]. CMD may also coexist with obstructive coronary disease, potentially explaining persistent angina in up to a third of patients following percutaneous coronary intervention (PCI) [2]. Higher ischaemia burden is associated with worse clinical outcomes, including myocardial infarction and mortality [3]. In patients with ischaemia and non-obstructive coronary artery disease (INOCA), isolated CMD independently predicts poorer outcomes, with nearly a fourfold increase in mortality and a fivefold increase in major adverse cardiovascular events (MACE) [4,5,6].

MACE is used throughout this review as reported by the cited studies; however, its constituent endpoints vary considerably between studies, commonly including cardiovascular death, myocardial infarction, stroke, unplanned revascularisation, and hospitalisation for angina or heart failure. Importantly, MACE is not specific to plaque-rupture-related events, as several of its components, such as stroke and heart failure hospitalisation, may occur independently of coronary plaque rupture.

The pathophysiological mechanisms underlying increased MACE in patients with obstructive coronary artery disease (CAD) coexisting with CMD, and in those with CMD-driven INOCA, remain incompletely understood. As MACE often can result from vulnerable plaque (VP) rupture or erosion, it is biologically plausible that CMD and plaque vulnerability could be mechanistically linked. Emerging evidence suggests that vulnerable epicardial plaques may release inflammatory and vasoactive mediators that act on downstream resistance arterioles, potentially inducing or worsening CMD. Conversely, CMD-related alterations in coronary flow and shear stress may influence plaque inflammation and stability, establishing a bidirectional coronary–microcirculatory “crosstalk”. This framework linking epicardial plaque biology and microvascular dysfunction offers a parsimonious explanation for these overlapping clinical observations, consistent with the principle of Occam’s razor.

The purpose of this narrative review is to integrate mechanistic, imaging, physiological, and clinical evidence to help explain how CMD and VP biology may interact to promote ischaemia and adverse cardiovascular outcomes. Specifically, it aims to

(1) Consolidate current evidence supporting the bidirectional relationship between CMD and VP;
(2) Critically appraise studies that inform this mechanistic link;
(3) Explore the clinical and therapeutic implications of recognising CMD and VP as dynamically interconnected disease processes.

This framework is intended to provide a conceptual foundation for future mechanistic and interventional studies rather than prescriptive clinical guidance.

## 2. Challenges Predicting Vulnerable Plaque Rupture

Vulnerable plaque (VP) is pathologically defined as an atherosclerotic plaque at high risk of rupture or erosion and subsequent thrombosis. Our fundamental understanding of the morphological features associated with plaque vulnerability derives largely from histopathological and autopsy studies [7]. Coronary imaging aims to identify these features in vivo through imaging surrogates, often termed high-risk plaque features; however, these surrogates approximate rather than confirm the underlying pathology, and their concordance with true histological vulnerability remains imperfect. Advanced coronary imaging modalities, including intravascular ultrasound (IVUS), optical coherence tomography (OCT), near-infrared spectroscopy (NIRS) and CT coronary angiography (CTCA), can identify anatomical features of VP but remain limited predictors of plaque rupture and adverse cardiovascular events [8,9,10]. Plaque behaviour is dynamic, as rupture, erosion, and healing may occur silently, often progressing to chronic coronary syndrome rather than ACS [11,12,13]. Non-obstructive plaques contribute substantially to MACE despite being under-recognised clinically [14]. Anatomical imaging alone is insufficient to guide pre-emptive intervention strategies. In the PREVENT study, targeting non-obstructive plaques based on imaging high-risk features alone yielded a number-needed-to-treat value of 88 to prevent one cardiac event or death, underscoring the limitations of anatomy-based ‘plaque sealing’ approaches applied in isolation [15]. Autopsy studies have consistently shown that approximately half of fatal coronary thrombotic events arise from non-obstructive plaques (<50% diameter stenosis), highlighting the limited discriminatory value of angiographic severity alone [14]. Collectively, these data highlight the need for complementary strategies incorporating haemodynamic, inflammatory, and biological markers of plaque activity and stability (Table 1).

## 3. Coronary Microvascular Dysfunction

Despite advances in revascularisation, ischaemia frequently persists, often due to CMD, a heterogeneous and under-recognised condition. CMD is not a single disease entity but comprises distinct pathophysiological subtypes [16]:

- Type 1: CMD without myocardial disease or obstructive CAD (microvascular angina);
- Type 2: CMD in the context of myocardial disease;
- Type 3: CMD coexisting with obstructive CAD;
- Type 4: Iatrogenic CMD (e.g., following PCI/coronary artery bypass grafting).

Invasive coronary physiology further refines this classification into structural CMD (low CFR, high IMR) and functional CMD (low CFR, low IMR), deepening our understanding of its heterogeneity (Figure 1) [17]. Contemporary guidelines emphasise comprehensive invasive coronary functional testing to assess impaired vasodilatory capacity, microvascular spasm, epicardial coronary spasm, and mixed vasomotor phenotypes [18]. Therefore, alongside invasive assessment of CFR and microvascular resistance, vasoreactivity testing with intracoronary acetylcholine has an important role in selected patients with suspected ischaemia with INOCA. This approach enables physiological endotyping into microvascular angina associated with CMD (structural or functional), epicardial vasospastic angina, microvascular vasospastic angina, combined vasomotor phenotypes, and, importantly, non-cardiac chest pain when coronary functional testing is normal.

Structural and functional CMD endotypes have different underlying pathophysiologies and may relate differently to plaque biology, myocardial ischaemia, and treatment response, although this remains largely unexplored in human studies. Structural CMD, characterised by microvascular rarefaction, arteriolar remodelling, and elevated IMR, may reflect a more fixed, chronic microvascular substrate, potentially paralleling established, higher-burden atherosclerotic disease, and may be more responsive to mechanical or flow-redistributive interventions such as the coronary sinus reducer (discussed further below). In contrast, functional CMD, characterised by vasomotor and endothelial/metabolic dysfunction and with normal microvascular resistance at rest, may instead reflect a more dynamic, reversible state, potentially more responsive to pharmacological modulation. Whether structural and functional CMD are differentially associated with distinct plaque phenotypes, or whether they respond differently to VP-directed or crosstalk-targeted therapies, has not been directly investigated, and these proposed distinctions should be regarded as hypothesis-generating rather than established, though it is increasingly unlikely that CMD should be treated as a single homogeneous condition.

The high prevalence of CMD helps explain why up to 65% of patients with stable angina who undergo angiography are found to have no obstructive coronary disease [19]. Notably, 1,218 patients of whom 66% were female were excluded from the ISCHEMIA trial (14% of those screened) due to the absence of obstructive disease despite having moderate-to-severe ischaemia, highlighting a substantial burden of likely undiagnosed type 1 CMD [20].

The relationship between CMD and myocardial ischaemia is complex and varies across CMD subtypes and clinical phenotypes. CMD can act as a primary driver of ischaemia when impaired coronary flow reserve is insufficient to meet myocardial oxygen demand, as seen in type 1 CMD. Conversely, recurrent ischaemic insults arising from epicardial coronary disease, myocardial infarction, cardiomyopathy, microvascular spasm, revascularisation, or distal microembolisation can induce or exacerbate CMD through endothelial injury, inflammation, fibrosis, and remodelling. In acute coronary syndromes, patients who develop CMD, primarily due to microvascular obstruction from distal embolisation, exhibit markedly worse outcomes, with reduced coronary vasodilator capacity, persistent microvascular ischaemia, adverse ventricular remodelling, heart failure, and death [21,22,23]. However, in the absence of effective targeted therapies, CMD is often overlooked in routine clinical practice and remains underdiagnosed.

Distinguishing the dominant CMD mechanism has clinical relevance. Type 3 CMD may explain persistent angina following revascularisation for obstructive coronary artery disease, while revascularisation itself may paradoxically induce iatrogenic type 4 CMD, potentially limiting symptomatic benefit. Although recognition of CMD can support symptom-directed therapy and improve quality of life, current treatments are largely extrapolated from epicardial coronary disease and lack robust evidence of efficacy in CMD [24]. Therefore, CMD remains a major unmet need in the management of ischaemia.

## 4. The Ischaemia Paradox: Why Does Treating Ischaemia Alone Fail?

Patients with a greater ischaemia burden face higher risks of myocardial infarction and death, making ischaemia an important prognostic marker of both plaque rupture and MACE, even in the absence of obstructive CAD [3,19]. However, revascularising all ischaemia-inducing lesions in patients with stable CAD treated with optimal medical therapy does not significantly reduce mortality or future myocardial infarctions [20,25].

This raises a critical question: Why does treating ischaemia not translate into improved outcomes? And more fundamentally, how is ischaemia linked to plaque rupture and MACE?

One explanation is that ischaemia persists despite revascularisation. Up to one-third of patients have residual ischaemia post-PCI, often due to coexisting CMD which revascularisation does not address and may even exacerbate [2]. In this context, solely treating epicardial stenoses may leave the true cause of ischaemia untouched.

Another possibility is that ischaemia is not the primary driver or causally related to plaque rupture events, but rather a surrogate marker for underlying high-risk VP, which may be either obstructive or non-obstructive. VP can release inflammatory and vasoactive mediators that trigger CMD and downstream ischaemia without necessarily causing significant luminal stenosis. As a result, revascularising the ischaemic territory without addressing the underlying VP fails to prevent future plaque rupture and myocardial infarction.

Together, these observations suggest that persistent ischaemia, residual VP, and CMD may be mechanistically interlinked rather than independent targets, raising the possibility of a self-perpetuating cycle that neither revascularisation nor VP-directed therapy alone can fully interrupt. This redefines the therapeutic target from ischaemia itself to the underlying biology driving it, a possibility explored in detail below.

## 5. The Bidirectional Coronary to Microcirculatory Crosstalk Hypothesis

The bidirectional crosstalk hypothesis describes reciprocal interactions between VP and CMD, whereby shared inflammatory, metabolic, and haemodynamic mechanisms allow each process to amplify the other. Although traditionally considered distinct, emerging mechanistic and imaging data suggest these processes may be linked through common biological and haemodynamic pathways (Figure 2).

Inflammatory injury, endothelial dysfunction, and plaque destabilisation are well-established drivers of atherosclerosis, while altered coronary haemodynamics are known to influence plaque stability. Building on this, we propose a dynamic, two-way model in which plaque-derived inflammatory mediators and haemodynamic forces may impair downstream microvascular function, while CMD-related alterations in coronary flow and shear stress may, in turn, exacerbate plaque inflammation, progression and vulnerability. This framework proposes reframing VP and CMD as potentially mutually reinforcing components of coronary disease, rather than parallel independent processes. Notably, no randomised trials have tested whether improving CMD or coronary flow reserve can stabilise plaque or reduce plaque-related events, highlighting an important gap for future mechanistic and interventional studies.

## 6. Mechanistic Pathways Linking CMD to Plaque Destabilisation

CMD arises from diverse mechanisms, including cardiomyopathic changes, arteriolar remodelling, capillary rarefaction, vasomotor and endothelial dysfunction, extravascular compression, obstructive epicardial disease, and coronary recanalisation [26]. The links between CMD and VP destabilisation can be broadly grouped into haemodynamic, inflammatory, metabolic, and neurohumoral/diastolic pathways. Importantly, these mechanisms interact dynamically rather than acting in isolation, with their relative contributions varying across disease stages and patient phenotypes. This framework integrates these overlapping processes into a cohesive model that reflects the complexity of coronary pathophysiology.

## 7. Haemodynamic Pathways

CMD promotes a low-flow coronary state through increased microvascular resistance, capillary rarefaction, impaired vasodilatory capacity, and reduced CFR. This may manifest as sluggish coronary flow on angiography (coronary slow flow phenomenon) and prolonged transit times during invasive bolus thermodilution testing [27].

CMD-associated low coronary flow is linked to a worse prognosis, and computational fluid dynamics studies demonstrate that reduced coronary flow lowers endothelial shear stress (ESS), creating a proatherogenic milieu that accelerates VP progression [28,29]. CT and intracoronary imaging studies show that low ESS promotes plaque growth, necrotic core expansion, and constrictive remodelling, leading to luminal narrowing [30,31]. Low ESS downregulates vasculoprotective pathways while upregulating pro-inflammatory and pro-thrombotic signalling, including increased endothelial permeability to LDL and enhanced retention of inflammatory cells such as macrophages, which due to flow stagnation have a longer residence time at the site of plaque [30].

As plaques enlarge, their composition evolves and influences internal plaque structural stress (PSS), particularly across the fibrous cap [32]. High-risk plaques exhibit elevated PSS due to large necrotic cores and thin caps, with rupture occurring when PSS exceeds the mechanical strength of the fibrous cap. Plaque progression, often originating in regions of diffusely low ESS, leads to focal luminal narrowing; at this site, local flow acceleration through the narrowed lumen generates high ESS at the plaque throat even as flow remains reduced across the vessel as a whole [33,34,35,36]. These elevated frictional forces may further destabilise plaques by exacerbating cap thinning and tipping the mechanical balance in favour of rupture under high PSS forces, helping explain why rupture commonly localises to the upstream portion of atheroma [32].

## 8. Diastolic and Neurohumoral Pathways

Heart failure with preserved ejection fraction (HFpEF) is increasingly recognised not merely as a haemodynamic disorder but as a condition associated with a significantly elevated risk of MACE. Therapies that improve HFpEF, such as sodium–glucose cotransporter-2 (SGLT2) inhibitors, have demonstrated reductions in cardiovascular events, though the underlying mechanisms reducing plaque rupture events remain incompletely understood [37]. Evidence shows that improvements in left ventricular diastolic function may alleviate CMD, a condition present in up to 75% of patients with HFpEF [38]. CMD is associated with recurrent ischaemia, microinfarctions, and myocardial fibrosis and may both result from and contribute to impaired myocardial relaxation driving HFpEF [39].

Coronary forward flow is determined by two dominant waves; the first is the early forward compression wave caused by left ventricular ejection, and the second is the backward expansion wave (BEW) caused by microcirculatory decompression in early diastole as left ventricular relaxation draws blood from the coronary artery into the microvasculature [40]. As coronary flow occurs mainly in diastole, the BEW is the predominant driver of coronary flow. Diastolic impairment therefore reduces the BEW, lowering coronary flow, worsening CMD, and promoting plaque progression, while also reducing a patient’s capacity to augment flow during exercise (lower CFR), potentially exacerbating ischaemia and angina [38,40].

Structural CMD is frequently associated with systemic and exercise-induced hypertension, increasing afterload and myocardial oxygen demand, thereby worsening diastolic dysfunction [17]. Patients with structural CMD often exhibit elevated NT-proBNP levels, reflecting increased filling pressures and impaired relaxation. Impaired shear stress–mediated vasodilatation during exercise reflects reduced endothelial nitric oxide synthase (eNOS) activity, resulting in abnormal vascular tone in both coronary and peripheral beds. This is supported by reduced vasodilatation to acetylcholine, which normally acts via eNOS activation. Genetic polymorphisms leading to eNOS dysfunction are associated with CMD [41].

A recent large cross-sectional population study demonstrated that impaired systemic microvascular function assessed non-invasively in the forearm using a blood oxygen saturation recovery test, was associated with a higher cardiovascular risk profile and greater coronary artery calcium burden, suggesting that microvascular dysfunction parallels subclinical atherosclerosis and may serve as an early functional marker of cardiovascular risk [42].

Neurohumoral activation through the renin–angiotensin–aldosterone system (RAAS) is well known to increase cardiovascular risk, but it also contributes directly to CMD. Angiotensin II promotes oxidative stress, reduces nitric oxide bioavailability, and drives coronary microvascular rarefaction, while aldosterone enhances vascular inflammation and adverse remodelling [43,44]. In the WISE study, ACE inhibition with quinapril improved CFR and anginal symptoms in women with CMD [45].

## 9. Inflammatory Pathway

Inflammation is central to atherosclerosis and VP biology and provides a key mechanistic link to CMD. Atherosclerotic plaques release cytokines and chemokines that can act downstream on the microcirculation, impairing endothelial function, increasing vascular tone, and disrupting vasomotor regulation. Numerous inflammatory mediators are overexpressed in both obstructive and non-obstructive VP and could potentially contribute to CMD, such that ischaemia may act as a surrogate marker of VP even in patients with INOCA.

The interleukin-6 (IL-6) inflammatory axis plays a pivotal role in plaque instability and CMD. Elevated trans-myocardial IL-6 gradients are associated with increased microvascular resistance, impaired CFR, and intracoronary imaging features of VP [46,47]. Upstream mediators, including IL-1α and IL-1β, amplify vascular inflammation, are linked to adverse outcomes in ACS, and represent established therapeutic targets [48,49]. Targeting this pathway with biologic agents such as canakinumab (IL-1β inhibition) and ziltivekimab (IL-6 inhibition) offers a promising strategy to reduce plaque rupture events [50,51].

Endothelin-1 (ET-1), a potent vasoconstrictor and pro-inflammatory peptide, is overexpressed in unstable plaques and rises acutely after myocardial infarction [52,53]. Systemic Big ET-1 concentrations are associated with CTCA-defined high-risk plaque features [54]. Activation of the endothelin A receptor promotes coronary vasoconstriction and microvascular dysfunction; both elevated systemic ET-1 and greater trans-myocardial extraction correlate with increased coronary microcirculatory resistance [55,56]. The ET-1 antagonist zibotentan has been investigated as a potential treatment for microvascular angina [57]. Although it did not significantly improve exercise duration in the primary analysis, a substudy demonstrated improved subendocardial myocardial blood flow, suggesting a possible beneficial effect on CMD [57].

While inflammatory pathways clearly contribute to both CMD and plaque vulnerability, most evidence linking inflammation to CMD remains observational. Biomarker studies are heterogeneous, and clinical trials of anti-inflammatory therapies have not routinely incorporated microvascular endpoints. Table 2 summarises the broader range of inflammatory, oxidative, coagulation, endothelial, and metabolic biomarkers implicated in CMD; these represent associative findings from heterogeneous observational studies and should not be interpreted as evidence of causal or mechanistic proof.

Small mechanistic studies provide mixed signals, for example peri-procedural colchicine has been associated with higher CFR and resistive reserve ratio immediately after PCI, whereas IL-6 receptor inhibition with tocilizumab following MI showed no improvement in CFR [58,59]. Consequently, robust evidence that anti-inflammatory treatment improves CMD is lacking. Future studies should incorporate microvascular endpoints with MACE to clarify whether anti-inflammatory strategies directly modify CMD or exert indirect effects through plaque stabilisation.

**Table 2:** Biomarkers associated with coronary microvascular dysfunction [60,61].

Category	Biomarkers
Inflammation	C-reactive protein (CRP), high-sensitivity CRP (hs-CRP), interleukin-6 (IL-6), intracellular adhesion molecule-1 (ICAM-1), vascular adhesion molecule-1 (VCAM-1), suppressor of tumourigenicity-2 (ST2), peptidoglycan recognition protein (PGLYRP1), growth differentiation factor-15 (GDF-15), C-C motif chemokine 16 (CCL16), C-X-C motif chemokine 16 (CXCL16), tumour necrosis factor-α (TNF-α), galectin-4
Oxidative stress	Glutathione, malondialdehyde, superoxide dismutase, oxidised low-density lipoprotein (ox-LDL), myeloperoxidase (MPO)
Coagulation	Plasminogen activator inhibitor-1 (PAI-1), von Willebrand factor (vWF), tissue-type plasminogen activator (tPA), D-dimer, soluble urokinase plasminogen activator receptor (suPAR)
Endothelial dysfunction	Asymmetric dimethylarginine (ADMA), endothelin-1 (ET-1), vascular endothelial growth factor (VEGF), matrix metalloproteinase-9 (MMP-9), Klotho protein, CD40 ligand, renalase, serotonin
Metabolic	Homocysteine, low-density lipoprotein (LDL), apolipoprotein C (Apo-C), omega-3 fatty acids, uric acid

Table 2 summarises associative findings from heterogeneous studies reporting correlations between circulating biomarkers and objective measures of CMD, assessed using either invasive or non-invasive modalities. These data should be interpreted as evidence of association rather than definitive proof of mechanistic causality.

## 10. Metabolic Pathways

Metabolic dysregulation represents a key mechanism linking CMD with plaque vulnerability. Dyslipidaemia, insulin resistance, diabetes, and obesity promote endothelial dysfunction, impaired vasodilatory reserve, and oxidative stress, all of which contribute to CMD.

Low-density lipoprotein cholesterol (LDL) is pivotal in the development of CAD, and lowering LDL is a fundamental pillar in treating atherosclerosis. However, there is also strong evidence that elevated LDL is related to CMD [62]. LDL is an independent predictor of raised IMR and inversely correlates with CFR [63,64]. LDL and in particular oxidized LDL induces endothelial and microcirculatory dysfunction by decreasing nitric oxide bioavailability and via actions on lectin-like oxidized low-density lipoprotein-1 (LOX-1). High dose statin therapy in patients with elevated LDL also modestly improves CMD [65].

Diabetes is strongly associated with CMD, even in the absence of overt CAD, with lower CFR associated with increased glycated haemoglobin levels and the presence of albuminuria in diabetic patients [66,67]. Mechanisms include endothelial dysfunction with reduced nitric oxide bioavailability, accumulation of advanced glycation end products, oxidative stress, microvascular rarefaction, and perivascular fibrosis, leading to impaired autoregulation and recurrent ischaemia [68,69]. CMD in diabetic patients contributes not only to angina but also to silent ischaemia, recurrent microinfarctions, and adverse myocardial remodelling, which may accelerate plaque progression and destabilisation. Metabolic therapies, such as sodium–glucose cotransporter-2 (SGLT2) inhibitors, have shown benefit across several relevant domains, though evidence for each comes from separate studies rather than a unified demonstration. Empagliflozin improved anginal symptoms and exercise capacity in patients with type 2 diabetes and refractory angina [70]; preclinical work suggests a microvascular/nitric-oxide-mediated mechanism by which SGLT2 inhibition may protect against heart failure independently of glycaemic control [71], and separately, SGLT2 inhibitors were associated with favourable changes in coronary plaque phenotype, including increased fibrous cap thickness, in diabetic patients [72]. Whether these effects reflect a shared mechanism bridging microvascular and plaque biology remains to be established.

Adipose tissue-derived cytokines (“adipokines”) play key roles in vascular homeostasis. In obesity, an imbalance between pro-inflammatory adipokines (e.g., leptin, resistin) and the protective adipokines (e.g., adiponectin) promotes oxidative stress, endothelial dysfunction, and microvascular rarefaction [73]. Evidence indicates that excess adiposity is closely associated with subclinical diastolic dysfunction and reduced myocardial perfusion, reinforcing a mechanistic continuum linking obesity, diastolic impairment, and CMD [73].

In summary, a proposed self-perpetuating vicious cycle may emerge in which VP may promote CMD and myocardial ischaemia through the release of vasoactive molecules. This, in turn, may impair left ventricular relaxation, reduce coronary flow, and lower ESS at sites of plaque, potentially further accelerating plaque progression and the release of additional vasoactive mediators (Figure 2).

Could therapies that improve diastolic function and myocardial relaxation also restore microvascular function, enhance coronary flow, and slow plaque progression, potentially reducing cardiovascular events related to plaque rupture? This remains speculative, but if confirmed, it would offer a plausible unifying explanation for the pleiotropic benefits observed with SGLT2 inhibitors, and would support CMD as a potential modifiable contributor to atherosclerotic disease rather than solely a downstream consequence of it. This conceptual framework may help guide future research into therapeutics targeting the proposed crosstalk cycle, with potential relevance to CMD, HFpEF, VP progression, and long-term cardiovascular risk. However, this hypothesis requires confirmation through prospective, mechanistically linked studies.

## 11. Current Tools for Assessing Plaque–Microcirculatory Crosstalk

To disrupt plaque–microcirculatory crosstalk, it must first be identifiable and quantifiable. However, routine assessment of CMD remains uncommon despite the availability of invasive and non-invasive tools.

Invasive coronary function testing using thermodilution or Doppler techniques enables derivation of CFR, IMR, and MRR. Comprehensive invasive functional assessment can be extended to include intracoronary acetylcholine or ergonovine provocation testing, enabling identification of epicardial and microvascular vasospasm, endotypes increasingly recognised within the INOCA spectrum and incorporated into contemporary ESC-endorsed diagnostic algorithms [18,74]. Despite their diagnostic value, routine use remains limited by procedural complexity, cost, and the expertise required for acquisition and interpretation. As awareness of the clinical and prognostic significance of CMD increases, broader adoption of such testing in the catheterisation laboratory is expected. The 2024 ESC Guidelines on Chronic Coronary Syndromes assign invasive coronary function testing a Class I, Level B recommendation for patients with non-obstructive coronary arteries and persistent angina despite optimal therapy [74]. Reflecting this, invasive coronary function testing represents the current reference standard for definitive diagnosis and mechanistic endotyping of CMD, given its unique ability to directly quantify CFR, IMR, and vasoreactivity. Invasive intracoronary imaging can also characterise anatomical high-risk plaque features. However, routine three-vessel intracoronary imaging is unlikely to be widely adopted in patients with non-obstructive disease, given the additional procedural risk and time added to the procedure.

In practice, non-invasive investigations are typically undertaken first, given their wider availability, lower cost, and risk. Consequently, non-invasive assessment of plaque vulnerability and microvascular function will remain central to the initial evaluation of patients with suspected CMD. Among non-invasive modalities, positron emission tomography (PET) is a well-validated, quantitative technique for assessing myocardial blood flow and CFR, with additional ability to assess systemic and coronary plaque inflammatory activity [75,76]. However, PET is costly and not widely available. Quantitative myocardial perfusion cardiac MRI is increasingly accessible and provides valuable diagnostic and prognostic information [77]. The 2021 Multisociety Chest Pain Guideline, endorsed in the 2025 American Heart Association (AHA) scientific statement on nonobstructive CAD, assigns a Class IIa recommendation to both stress CMR and PET as the primary non-invasive functional tests for evaluating suspected INOCA prior to invasive physiologic assessment [78].

Of the available physiological indices, CFR is the parameter most consistently and robustly linked to cardiovascular prognosis, with reduced CFR independently associated with increased all-cause mortality and MACE across a broad range of cardiovascular conditions [79]. Transthoracic Doppler echocardiography (TDE) of the distal left anterior descending artery offers a radiation-free, low-cost, bedside alternative for estimating CFR. In patients with INOCA, non-invasive TDE-derived CFR correlates significantly with invasively measured CFR, supporting TDE as a reliable, accessible tool for early identification of CMD in appropriately selected patients, although image quality and operator expertise remain important limitations [80].

Exercise stress echocardiography (ESE) offers a more direct, widely available means of assessing inducible myocardial ischaemia, without ionising radiation or iodinated contrast exposure. A recent meta-analysis of 104 studies (16,824 symptomatic patients) reported pooled sensitivity and specificity of 81% and 85% for stress echocardiography, compared with 82% and 74% for stress myocardial single-photon emission computed tomography (SPECT) [81]. Given its accessibility and established role in evaluating suspected obstructive CAD, ESE represents a practical first-line or complementary non-invasive option, particularly when functional rather than purely anatomical information is required. A positive ESE in the absence of obstructive epicardial disease may be suggestive of underlying CMD. Similarly in patients with unobstructed coronary arteries, a simple exercise tolerance test demonstrating ischaemia can also suggest underlying CMD, although sensitivity remains low at approximately 35% [82].

CTCA, often the first-line investigation for stable chest pain, provides a practical platform for assessing plaque–microcirculatory interactions. CTCA enables detailed assessment of plaque morphology and adverse anatomical characteristics while also incorporating computational fluid dynamics (e.g., CT-derived fractional flow reserve and wall shear stress mapping) [83]. CTCA additionally allows quantification of the coronary artery calcium score, a well-established prognostic marker for future plaque rupture events. Contemporary CT-based techniques enable simultaneous evaluation of coronary perivascular inflammation via the fat attenuation index (FAI), a strong predictor of future MACE [84]. These complementary approaches provide both anatomical and functional insights into plaque vulnerability and have demonstrated independent and additive long-term prognostic value [85]. Further, ongoing development of CT-based microvascular resistance ratio (CT-MRR) aims to provide a comprehensive, non-invasive assessment of microvascular physiology, although further validation against invasive standards is required [86]. In the near future, a single CTCA acquisition may provide an integrated assessment of epicardial and microvascular physiology, plaque vulnerability, and vascular inflammation, representing a potentially ideal risk stratification tool for CAD.

Taken together, a stepwise diagnostic pathway is reasonable in practice, as outlined in the EAPCI consensus statement on INOCA [18]. Following clinical evaluation (history, risk factors, physical exam, ECG), patients with suspected CMD undergo non-invasive testing, with the choice between anatomical (CTCA) and functional (ESE, TTDE, quantitative stress CMR, or PET) assessment guided by pre-test likelihood (low risk favouring anatomical, high risk favouring functional), local expertise, and whether viability assessment is needed. The consensus document notes either may reasonably come first, and they can be combined rather than sequenced strictly. Where non-obstructive disease is confirmed but symptoms and functional testing remain suggestive of a microvascular or vasospastic cause, the consensus recommends invasive functional coronary angiography as the reference standard for definitive endotyping: guidewire-based adenosine testing (FFR, CFR, IMR) identifies coronary microvascular dysfunction, while intracoronary acetylcholine provocation is required to distinguish microvascular spasm from epicardial vasospasm and from combined disease, since endothelium-dependent testing cannot currently be performed non-invasively.

Despite a wide array of inflammatory biomarkers being associated with cardiovascular risk, three markers currently have practical value for assessing the cardiovascular inflammatory state in current clinical practice: LDL, high-sensitivity C-reactive protein (hs-CRP), and lipoprotein(a) (Lp(a)). LDL assessment and treatment are routine in both primary and secondary prevention, while hs-CRP and Lp(a) remain underutilised despite strong evidence linking their elevation to residual inflammatory and atherothrombotic risk, and their recent inclusion in guidelines for the diagnostic work-up of patients with chronic coronary syndromes and dyslipidaemia [74,87]. Elevated hs-CRP, in particular, is a powerful predictor of recurrent cardiovascular events and cardiovascular death, often outperforming LDL in secondary prevention cohorts [88]. Importantly, these three biomarkers confer additive risk: patients with concurrent elevations in LDL, hs-CRP, and Lp(a) demonstrate the highest likelihood of adverse outcomes [89].

In the setting of CMD, elevation of these biomarkers signals combined lipid, and inflammatory risk may increase plaque–microcirculatory crosstalk and subsequent plaque rupture, underscoring the need for more aggressive preventive strategies. Therapeutically, effective interventions are available not only for elevated LDL but also for raised hs-CRP and inflammation (such as colchicine in secondary prevention) and targeted Lp(a)-lowering therapies are currently in late-phase development, with the phase 3 HORIZON trial expected to expand future treatment options [90,91].

At present, no intervention has been shown to alter outcomes specifically by disrupting plaque–microcirculatory crosstalk, and this framework should be regarded as research-oriented rather than practice-changing. In a patient with stable angina, INOCA, and confirmed CMD, current management therefore remains guideline-directed; risk factor control and anti-anginal therapy agents as per existing evidence rather than crosstalk-specific. The diagnostic tools described above are best positioned, for now, as a means of enriching future trial populations by CMD and plaque phenotype, rather than as a decision aid for individual patients.

## 12. Evidence Gaps and Future Research Needs

While our proposed bidirectional crosstalk framework is supported by converging mechanistic and correlative data, direct interventional or longitudinal evidence confirming causal interplay between CMD and plaque progression is currently lacking (Table 3).

Numerous biomarkers have been associated with significant epicardial atheroma, inflammation, and VP morphology, though few have reached clinical application in CMD. For example, in hypertensive patients, Wang et al. demonstrated that the triglyceride–glucose (TyG) index (surrogate marker of insulin resistance) was independently associated with functionally significant coronary stenosis (CT-FFR ≤ 0.80), as well as adverse plaque features and higher perivascular inflammation on CT, linking metabolic dysregulation with both anatomical and functional vulnerability [92]. Data that are currently lacking and would be of particular interest include determining whether such metabolic and inflammatory biomarkers are similarly associated with, or contribute to CMD.

Recent data suggest that plaque composition may influence the severity of ischaemia. In a multicentre study using non-contrast T1-weighted coronary MRI, Sugane et al. demonstrated that high-intensity plaques were independently associated with lower distal coronary artery FFR in patients with stable coronary artery disease [93]. As the generation of a reduced FFR requires an intact hyperaemic microvascular response, these findings primarily support a link between plaque morphology and epicardial haemodynamic significance. Whether high-risk plaque characteristics are also associated with downstream microvascular dysfunction, or whether CMD modifies the physiological expression of such plaques, remains to be determined.

Trials in the ACS population would also be particularly valuable. Approximately half of recurrent myocardial infarctions following an index ACS arise from non-culprit lesions that are non-obstructive at the time of presentation [11]. The PECTUS-obs study demonstrated that such FFR-negative, non-culprit plaques frequently exhibit high-risk morphological characteristics on OCT and were associated with worse outcomes driven primarily by unplanned revascularisation [94]. Furthermore, VP features were notably more common in the female cohort [94]. As women are also predisposed to CMD, investigating the relationship between CMD and non-culprit plaque progression in post-ACS populations could provide important insights into sex-specific drivers of residual cardiovascular risk. Importantly, pressure-wire assessment early after ACS may underestimate lesion severity because MI-related CMD can impair hyperaemic response, resulting in falsely negative FFR values. This limitation further highlights the importance of assessing microvascular function alongside epicardial physiology when evaluating non-culprit plaques and exploring plaque–microcirculatory crosstalk in post-ACS populations.

Recent work in diabetic patients with non-obstructive coronary artery disease demonstrated that a novel global CT-derived fractional flow reserve gradient, which summates the ΔCT-FFR across all lesions in the major epicardial arteries (GΔCT-FFR) independently predicted MACE [95]. These findings highlight that even subtle epicardial haemodynamic abnormalities can confer prognostic risk beyond anatomical plaque assessment, supporting the concept of integrated plaque–flow interactions. As diabetic patients are particularly prone to microvascular dysfunction, future studies exploring the interplay between these epicardial haemodynamic forces and CMD would be of particular interest in refining risk prediction and identifying therapeutic targets.

While the bidirectional crosstalk hypothesis provides an integrative framework linking plaque biology and microvascular dysfunction, its generalisability across patient populations remains uncertain. CMD encompasses heterogeneous phenotypes ranging from structural microvascular rarefaction to functional vasomotor dysfunction, each influenced by distinct risk profiles, comorbidities, and underlying myocardial disease. These pathophysiological differences may modify both the strength and direction of the proposed interactions.

Future studies should therefore stratify patients by CMD subtype and clinical context to better define which mechanisms predominate and in whom targeted interventions may be most effective. The present framework remains hypothesis-generating, underscoring the need for prospective studies designed to jointly quantify microvascular function, CMD phenotype, plaque evolution, and clinical outcomes to confirm or refute bidirectionality. Two study designs would be particularly well suited to testing this. First, longitudinal cohort studies assessing whether baseline CMD independently predicts subsequent progression of specific VP features, after adjustment for conventional atherosclerotic risk factors, and conversely whether baseline plaque vulnerability predicts subsequent deterioration of coronary microvascular function would provide substantially stronger evidence for bidirectional causality than the cross-sectional associations available to date. Second, mechanism-based intervention trials, in which patients are simultaneously phenotyped for plaque characteristics and CMD and then randomised to targeted therapies, would offer a considerably stronger test of the proposed bidirectional model than observational associations alone.

## 13. Emerging Technologies Leveraging the Bidirectional Hypothesis

Novel coronary catheters now allow direct measurement of trans-plaque gradients of biomarkers released from plaque into the boundary layer allowing valuable insights into local inflammatory processes within the coronary vasculature microenvironment [96]. In the future, such device measurements could be used to endotype VP at the point of care during coronary catheterisation. The ongoing Biopattern study (ClinicalTrials.gov NCT06259019) is an observational study that aims to assess trans-plaque coronary gradients of a range of biomarkers and their association with OCT-defined features of VP, and CMD using invasive coronary physiology at the time of biomarker sampling, and future adverse outcomes [97]. The study aims to discover and highlight new potential biomarkers that could be the target for new future therapeutics targeting VP, CMD, HFpEF and ultimately reduce VP rupture events in patients with CAD.

Patients with ischaemia in whom VP is hypothesised to induce CMD via vasoactive markers may have a significantly poorer prognosis and higher future MACE compared to patients with ischaemia secondary to a stable, flow-limiting epicardial lesion alone. Therefore, not all ischaemia may be the same and blindly revascularising all ischaemia-inducing coronary lesions will not necessarily reduce cardiovascular events, leading to the ischaemia paradox witnessed in the ISCHEMIA trial. Improved trial design, utilising this knowledge in a precision medicine approach that may subclassify ischaemia linked to VP, perhaps utilising a high-risk biomarker or “biosignature” surrogate for VP, could for the first time demonstrate revascularisation improves prognosis in patients with stable IHD.

Finally, the bidirectional crosstalk model may provide a better understanding of the pathophysiology of VP and possibly systemic and local coronary biomarkers may be the last piece of the puzzle defining a biosignature of VP at highest risk of rupture and subsequent ACS. Improving identification of high-risk VP endotypes by also using biomarker profiles, physiological indices of CMD and flow dynamics (e.g., ESS/PSS) could further unmask those plaques which would benefit greater from prophylactic “plaque sealing” with stents to reduce the “numbers-needed-to-treat” to lower MACE [15]. Furthermore, there may also be a beneficial “off-target” improvement in CMD and HFpEF.

## 14. Future Anti-Inflammatory Treatments for Vulnerable Plaque

Improved endotyping of VP could also highlight patients who would benefit from specific anti-inflammatory treatments. If VP are found to locally express or release specific cytokines or biomarkers, directly targeting those cytokines or pathways with monoclonal antibodies could facilitate precision medicine use of expensive biologics with cost-effectiveness advantages.

Several anti-inflammatory therapies have been evaluated in cardiovascular clinical trials, as summarised in Table 4. Collectively, these studies demonstrate that targeting inflammation can favourably modify cardiovascular outcomes across a spectrum of patient cohorts, from secondary prevention to acute coronary syndromes. At present, colchicine remains the only anti-inflammatory agent incorporated into guideline recommendations for secondary prevention. Multiple randomised controlled trials have demonstrated that inexpensive, low-dose colchicine exerts broad anti-inflammatory effects and reduces MACE in patients with stable coronary artery disease [98,99]. A recent meta-analysis of over 21,000 patients reported a 25% relative reduction in MACE with colchicine in secondary prevention populations, driven primarily by reductions in myocardial infarction, ischaemic stroke, and coronary revascularisation [100]. Notably, this benefit exceeded that observed in the landmark CANTOS trial and was achieved without any increase in adverse safety outcomes compared with placebo. In contrast, monoclonal antibody–based therapies such as canakinumab, while mechanistically potent, have been constrained by adverse effects, particularly an increased risk of fatal infection and sepsis limiting their clinical adoption [50].

Importantly, none of these trials directly evaluated the effects of anti-inflammatory therapy on coronary microvascular function, an area of considerable mechanistic and therapeutic uncertainty. Future studies integrating microvascular and plaque-specific endpoints are needed to determine whether modulation of inflammation can disrupt the proposed plaque–microcirculatory feedback loop.

## 15. Future Coronary Microvascular Dysfunction Treatments

There remains a paucity of high-quality data for the treatment of CMD, particularly large prospective studies demonstrating improvements in objective microvascular indices. Much of the available evidence derives from subgroup analyses of broader cardiovascular trials, introducing potential selection bias due to imbalanced patient characteristics. Moreover, many interventions have been evaluated in patients with suspected rather than objectively confirmed CMD, as well as rarely stratified by CMD phenotype, factors that likely dilute observed treatment effects and obscure phenotype-specific therapeutic benefits.

There are currently no approved or licensed treatments specific to CMD, and this may explain clinical nihilism, as diagnosing CMD will not lead to a meaningful change in patient management. Future clinical trials incorporating the bidirectional hypothesis and using holistic diagnostics encompassing CMD endotypes, may discover new treatment targets in the form of vasoactive molecules liberated from VP that induce CMD. Furthermore, direct treatments for VP may indirectly treat CMD by reducing vasoactive molecule release. Such disease modifying agents may not only be useful in treating CMD and microvascular angina, but may also play a role in the management of diastolic heart failure and reducing cardiovascular risk.

The recent, large, randomised WARRIOR trial (n = 2476) evaluated intensive medical therapy with pleiotropic agents (statin, ACE inhibitor or angiotensin receptor blocker (ARB), and low-dose aspirin) against usual care in women with INOCA [109]. The trial found no significant reduction in MACE and was neutral for key secondary endpoints, including quality of life and anginal symptoms. In both arms, MACE was primarily driven by hospitalisation for angina despite unobstructed coronaries. The cohort was heterogeneous in symptom severity, and objective invasive assessment of IMR, CFR, or CMD subtype was not performed. Notably, WARRIOR tested therapies targeting atherosclerotic risk (statin, ACE inhibitor/ARB, aspirin) rather than microvascular function directly. Treating atherosclerotic risk alone may be insufficient when CMD remains undefined, although the neutral result is equally consistent with these agents simply being ineffective for INOCA regardless of underlying CMD status. Future trials incorporating CMD phenotyping alongside microvascular-targeted therapy would be needed to directly test whether treating CMD influences MACE and anginal symptoms in INOCA.

In contrast, promising early results have emerged from trials evaluating the coronary sinus reducer in patients with objectively confirmed CMD using invasive coronary physiology [110,111,112,113]. A recent Phase II trial investigating the coronary sinus reducer in patients with INOCA, significant anginal symptoms (Canadian cardiovascular society (CCS) angina class III–IV), and objectively defined CMD using invasive coronary physiology demonstrated significant improvements in both anginal symptoms and invasive CMD indices, including CFR and acetylcholine-mediated coronary blood flow [110]. Participants were stratified into endothelium-dependent and endothelium-independent CMD phenotypes, allowing for mechanistic insight. A larger, single-arm study in 40 patients with similarly severe angina (CCS III–IV) and invasively confirmed CMD corroborated these findings, reporting significant improvements in CFR, IMR, and angina-related quality of life following reducer implantation [113]. The device functions by narrowing the coronary sinus to increase backward venous pressure, thereby recruiting subendocardial microvasculature, reducing microvascular resistance, and enhancing CFR. This mechanism is likely to be most effective in patients with structural CMD and elevated baseline IMR, highlighting the importance of accurate CMD phenotyping when selecting and evaluating targeted therapeutic strategies. Although these studies are limited by their non-randomised, uncontrolled design, together they highlight the critical importance of CMD phenotyping and inclusion of objectively confirmed CMD rather than presumed cases to accurately evaluate therapeutic efficacy. Together, these findings underscore the need for larger, rigorously phenotyped, and mechanistically guided trials to identify effective, targeted therapies for CMD and its contribution to residual cardiovascular risk. Results from the ongoing REMEDY-PILOT trial (NCT05492110), a randomised, double-blind, sham-controlled study of the coronary sinus reducer in INOCA patients with confirmed CMD, with changes in quantitative perfusion CMR as its primary endpoint and a nested invasive microvascular physiology sub-study, are anticipated imminently and should help address the current absence of randomised, controlled evidence in this area [114].

The bidirectional crosstalk hypothesis can potentially broaden the range of drugs initially designed to treat one pathology that could indirectly impact another. For example, SGLT-2 inhibitors improve outcomes for diastolic impairment and refractory angina through likely increased nitric oxide generation, but on top of symptomatic benefits they have also been associated with reduced systemic inflammation and improved VP characteristics (e.g., increase in fibrous cap thickness, decrease lipid arc and macrophage grade) and lower rates of MACE including plaque rupture events [70,71,72]. Similarly, lipid-lowering drugs are an important pillar of secondary prevention and plaque stabilisation. In post-MI patients the proprotein convertase subtilisin/kexin type 9 (PCSK9) inhibitor evolocumab is associated with stabilisation and regression of high-risk features of non-culprit plaque as determined by OCT features [115]. Separately, early use of a PCSK9 inhibitor post-PCI for ACS has also been associated with improved microcirculatory function (lower IMR, higher CFR) compared to control patients [116,117]. It is important to note that these plaque-stabilising and microvascular-improving effects have generally been demonstrated in separate cohorts rather than within the same study population, and do not yet constitute direct evidence that modifying one process interrupts the other. Nonetheless, the observation that a treatment known to improve plaque phenotype and reduce MACE may also improve microcirculatory function raises the possibility of ‘indirect’ therapeutic effects on CMD and HFpEF that merit prospective, mechanistically linked investigation [118].

## 16. Conclusions

The bidirectional crosstalk hypothesis offers a novel conceptual lens for understanding how inflammation, ischaemia, microvascular resistance, HFpEF, coronary flow, and shear stress may interact to influence the progression and rupture of VP and ultimately MACE. It helps explain how HFpEF and CMD may exert prognostically important effects through mechanisms of plaque instability and how therapies targeting these processes might reduce plaque rupture-related events. This hypothesis may lay the foundation for improved endotyping of VP and the development of multi-parametric risk assessments to predict VP rupture, ultimately enabling precision-tailored therapeutic strategies to reduce cardiovascular risk and treat CMD and diastolic dysfunction. The bidirectional crosstalk hypothesis offers a promising framework to inform integrated trial design and precision-medicine approaches. However, it should be regarded as a conceptual model rather than an established mechanism, as it requires prospective validation through studies that simultaneously assess plaque phenotype, coronary physiology, CMD endotype, myocardial ischaemia, and longitudinal cardiovascular outcomes.

## Figures and Tables

**Figure 1:**
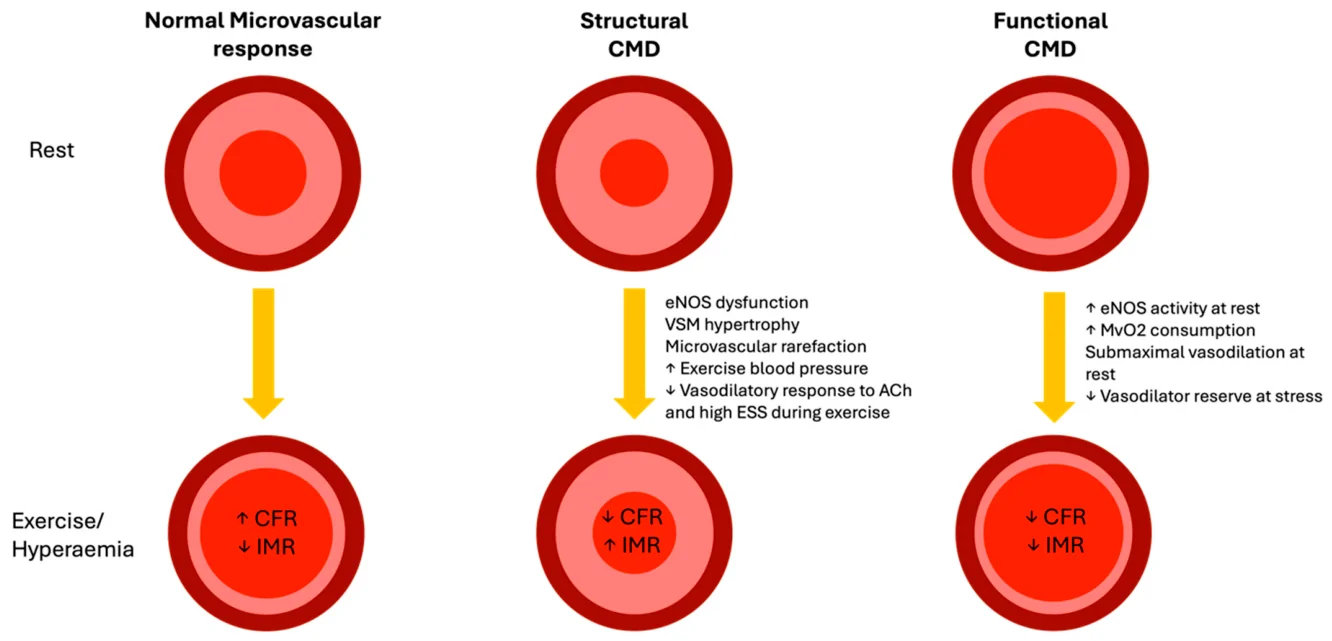
Haemodynamic endotyping of coronary microvascular dysfunction [17]. Coronary microvascular dysfunction (CMD) can be classified haemodynamically using coronary flow reserve (CFR) and the index of microcirculatory resistance (IMR) as described by Rahman et al. [17]. Normal microvascular function is defined by preserved CFR and low IMR. Structural CMD is characterised by reduced CFR and elevated IMR, indicating increased microvascular resistance that limits coronary flow augmentation during stress. Functional CMD is defined by reduced CFR and low IMR, reflecting impaired vasodilatory reserve despite low microvascular resistance. This classification supports diagnostic interpretation and may help guide targeted therapeutic strategies. Legend: CMD: coronary microvascular dysfunction, eNOS: endothelial nitric oxide synthase, VSM: vascular smooth muscle, Ach: acetylcholine, ESS: endothelial shear stress, MvO2: myocardial oxygen consumption, CFR: coronary flow reserve, IMR: index of microcirculatory resistance, ↑: increased, ↓: decreased.

**Figure 2:**
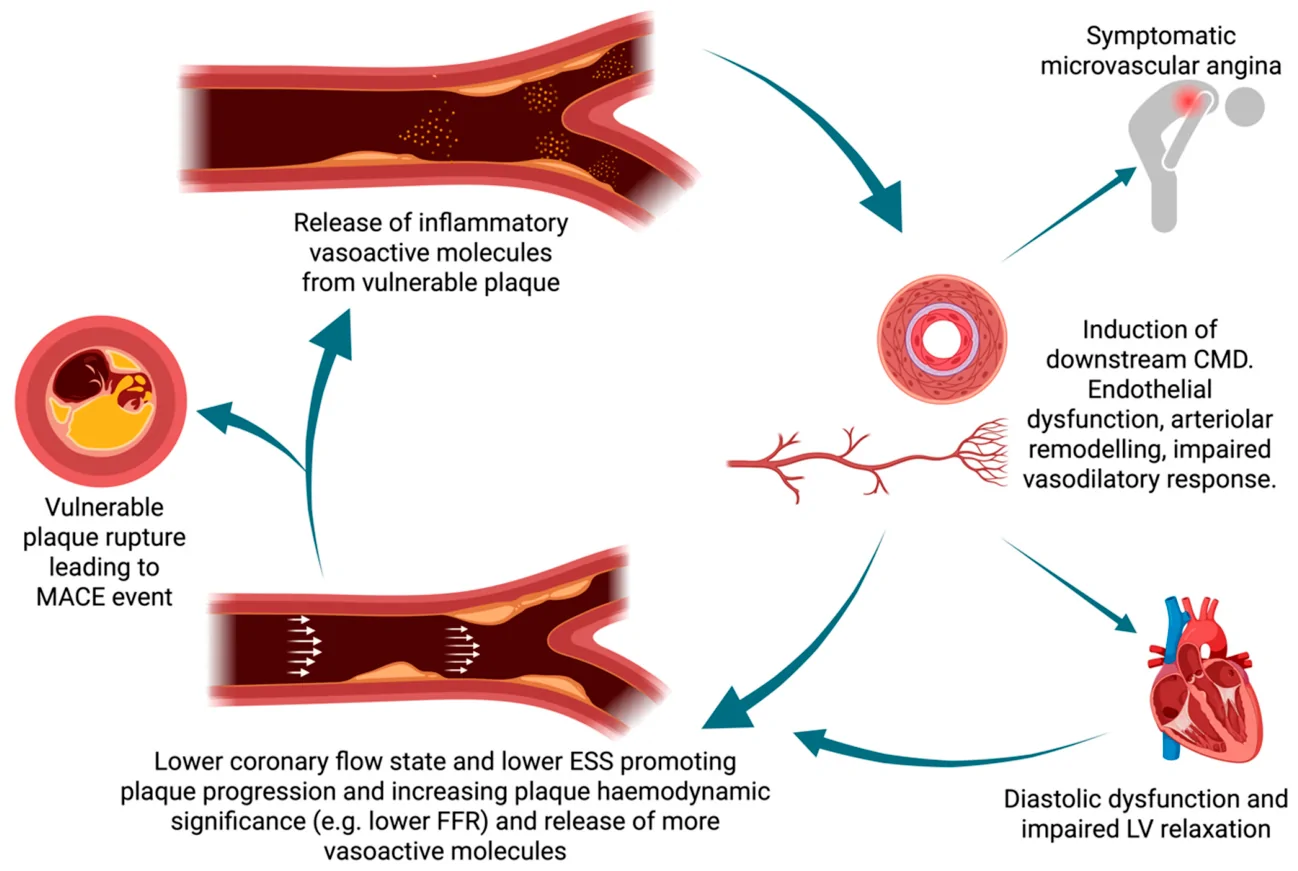
Central illustration: Bidirectional coronary to microvasculature crosstalk hypothesis. Vulnerable epicardial plaques are hypothesised to release inflammatory and vasoactive mediators capable of impairing downstream microvascular function, potentially contributing to CMD, angina, and diastolic dysfunction. CMD and diastolic dysfunction may together reduce coronary flow and ESS, potentially promoting further plaque progression, instability, additional release of vasoactive molecules, and ultimately plaque rupture. This proposed self-perpetuating cycle may underlie the link between ischaemia, CMD, and VP rupture, and highlights potential therapeutic targets beyond conventional revascularisation strategies. Legend: CMD = Coronary microvascular dysfunction, LV = left ventricle, ESS = endothelial shear stress, FFR = Fractional flow reserve, MACE = Major adverse cardiovascular event. Created in BioRender. Davenport, A. (2026) https://BioRender.com/5d9shu1 (accessed on 19 September 2026).

**Table 1:** Diagnostic modalities and parameters for coronary plaque characterisation and risk stratification.

Diagnostic Technique	Key Parameters
Anatomical imaging (invasive and non-invasive)	Thin cap fibroatheromas Plaque burden Minimal luminal area Negative remodelling
Intravascular near-infrared spectroscopy	Lipid core burden index (quantification of lipid core burden)
Functional assessment of haemodynamic significance	Invasive fractional flow reserve (FFR) Resting non-hyperaemic indices CT-derived FFR
Assessment of coronary microvascular function	Index of microcirculatory resistance (IMR) Coronary flow reserve (CFR) Microvascular resistance reserve (MRR)
Computational modelling and flow dynamics	Plaque structural stress (PSS) Endothelial shear stress (ESS)
Molecular and cellular plaque environment	Trans-coronary biomarker gradients Systemic inflammatory markers

**Table 3:** Evidence strength for each proposed step in the plaque–microcirculatory crosstalk hypothesis.

Step	Evidence Strength	Supporting Evidence	Key Limitation
1. Coronary plaque has anatomical features that confer vulnerability	Established	Thin cap fibroatheroma, large lipid core, positive remodelling, and low minimal luminal area validated on IVUS/OCT/NIRS and confirmed at autopsy and in PROSPECT [8,9,10,11,12,13,14]	These features alone poorly predict which plaques will actually rupture; PREVENT showed an NNT of 88 for anatomy-guided sealing [15]
2. Vulnerable plaque releases/expresses biomarkers into the coronary circulation	Strong observational	Direct trans-coronary sampling studies demonstrate measurable IL-6 and endothelin-1 gradients across plaque [46,47,55]	Sampling is cross-sectional; whether gradient magnitude tracks plaque instability over time is unknown
3. Plaque-derived biomarkers cause or induce CMD	Observational/mechanistically plausible, not causally established	Elevated trans-myocardial IL-6 and ET-1 correlate with impaired CFR and elevated IMR; ET-1 receptor activation is a well-characterised vasoconstrictor mechanism in vitro [46,47,55,56]	No study has isolated plaque-derived (vs. systemic) mediators as the specific cause of CMD in humans; association does not establish direction or causality
4. CMD is associated with poorer prognosis and a higher incidence of MACE, including myocardial infarction	Established for prognosis and MACE. A direct association with plaque rupture has not been established.	Isolated CMD independently predicts mortality and MACE [4,5,6].	Existing prognostic data use composite MACE, which is not synonymous with plaque-rupture events specifically; this distinction is not yet isolated in the literature
5. CMD leads to coronary plaque progression	Mechanistic only	CMD-related low coronary flow is associated with low ESS, which correlates with plaque growth in imaging/CFD studies [28,29,30,31]	No study has prospectively followed CMD-phenotyped patients to show accelerated plaque progression compared with matched controls without CMD
6. Treating CMD reduces plaque progression or VP events	No direct evidence	None identified	Genuine, untested gap: no trial has assessed plaque endpoints following CMD-targeted therapy (e.g., coronary sinus reducer trials report CFR/IMR/symptoms only)
7. Treating vulnerable plaque improves CMD	No direct evidence (indirect only)	SGLT2i and PCSK9i show plaque-stabilising and microvascular-improving effects, but in separate patient cohorts rather than the same study population	No trial has directly tested whether VP-targeted therapy improves CMD indices within one cohort

**Table 4:** Anti-inflammatory therapies evaluated in cardiovascular clinical trials.

Drug	Trial (Year)	Population	Outcome
Canakinumab	CANTOS (2017)	Previous MI, raised hs-CRP, n = 10,061	MACE reduction at 48 months, HR 0.85 (0.74–0.98) [50]
Anakinra	MRC-ILA (2015); VCU-ART3 (2020)	Post-NSTEMI, n = 182; Post-STEMI, n = 99	Significant hs-CRP reduction at 7 and 14 days, respectively [101,102]
Tocilizumab	ASSAIL-MI (2021)	Post-STEMI, n = 101	Significant increase in myocardial salvage index (cardiac MRI, 3–7 days) [103]
Ziltivekimab	RESCUE (2021)	CKD, raised hs-CRP, n = 264	Significant hs-CRP reduction at 12 weeks [104]
Interleukin-2	IVORY (2024)	Post-ACS, raised hs-CRP, n = 60	Reduced arterial inflammation (CT-PET) [105]
Colchicine	COLCOT (2019); LODOCO2 (2020)	Recent ACS, n = 4745; Chronic coronary disease, n = 5522	MACE reduction, HR 0.77 and 0.69, respectively [91,99]
Colchicine	CONVINCE (2024)	Recent stroke/TIA, n = 3154	No statistically significant MACE reduction, HR 0.84 (0.68–1.05) [106]
Methotrexate	CIRT (2018); TETHYS (2017)	Previous MI/multi-vessel CAD, n = 4786; Post-STEMI, n = 84	No significant MACE or infarct-size benefit [107,108]

MI = myocardial infarction; hs-CRP = high-sensitivity C-reactive protein; MACE = major adverse cardiovascular event; HR = hazard ratio; NSTEMI = non-ST-elevation myocardial infarction; STEMI = ST-elevation myocardial infarction; MRI = magnetic resonance imaging; CKD = chronic kidney disease; ACS = acute coronary syndrome; CT-PET = computed tomography and positron emission tomography; TIA = transient ischaemic attack.

## Data Availability

No new data were created or analysed in this study. Data sharing is not applicable to this article.

## Abbreviations

ACS	Acute coronary syndrome
BEW	Backward expansion wave
CAD	Coronary artery disease
CFR	Coronary flow reserve
CMD	Coronary microvascular dysfunction
CTCA	Computed tomography coronary angiography
ESS	Endothelial shear stress
ET-1	Endothelin-1
ET_A_	Endothelin A receptor
FFR	Fractional flow reserve
HFpEF	Heart failure with preserved ejection fraction
hs-CRP	High-sensitivity C-reactive protein
IL-1*α*	Interleukin-1*α*
IL-1β	Interleukin-1β
IL-6	Interleukin-6
IMR	Index of microcirculatory resistance
INOCA	Ischaemia with no obstructive coronary arteries
IVUS	Intravascular ultrasound
LDL	Low-density lipoprotein
LOX-1	Lectin-type oxidized low-density lipoprotein receptor 1
MACE	Major adverse cardiovascular event
MRR	Microvascular resistance reserve
NIRS	Near-infrared spectroscopy
OCT	Optical coherence tomography
PCI	Percutaneous coronary intervention
PCSK9	Proprotein convertase subtilisin/kexin type 9
PSS	Plaque structural stress
SGLT-2	Sodium–glucose co-transporter-2
VP	Vulnerable plaque
